# Cardiotoxicity of Iron and Zinc and Their Association with the Mitochondrial Unfolded Protein Response in Humans

**DOI:** 10.3390/ijms25179648

**Published:** 2024-09-06

**Authors:** Vid Mirosevic, Tomo Svagusa, Natalija Matic, Kresimir Maldini, Mario Siljeg, Davor Milicic, Hrvoje Gasparovic, Igor Rudez, Ana Sepac, Lucija Gojmerac, Ana Kulic, Petra Bakovic, Filip Sedlic

**Affiliations:** 1 Department of Pathophysiology, School of Medicine, University of Zagreb, 10000 Zagreb, Croatia; vid.mirosevic@gmail.com (V.M.); bakovicpetra@gmail.com (P.B.); filip.sedlic@mef.hr (F.S.); 2 Department of Cardiovascular Diseases, Dubrava University Hospital, 10000 Zagreb, Croatia; 3 Department of Development and Water Management, Croatian Waters, 10000 Zagreb, Croatia; nmatic@voda.hr; 4 Josip Juraj Stossmayer Water Institute, 10000 Zagreb, Croatia; 5 School of Medicine, University of Zagreb, 10000 Zagreb, Croatia; davormilicic01@gmail.com (D.M.);; 6 Department of Cardiovascular Diseases, University Hospital Centre Zagreb, 10000 Zagreb, Croatia; 7 Department of Surgery, School of Medicine, University of Zagreb, 10000 Zagreb, Croatiarudi@kbd.hr (I.R.); 8 Department of Cardiac Surgery, University Hospital Centre Zagreb, 10000 Zagreb, Croatia; 9 Department of Cardiac and Transplant Surgery, Dubrava University Hospital, 10000 Zagreb, Croatia; 10 Department of Pathology, School of Medicine, University of Zagreb, 10000 Zagreb, Croatia; 11 Ljudevit Jurak Department of Pathology, Sestre Milosrdnice University Hospital Centre, 10000 Zagreb, Croatia; 12 Department of Oncology, University Hospital Centre Zagreb, 10000 Zagreb, Croatia

**Keywords:** heart failure, cardiomyopathy, trace elements, mitochondrial unfolded protein response, oxidative stress

## Abstract

This study was designed to examine the association between myocardial concentrations of the trace elements Cu, Fe, Mn, Mo, and Zn and the expression of mitochondrial unfolded protein response (UPRmt) elements and the age of patients who received heart transplantation or a left-ventricular assist device (ageHTx/LVAD). Inductively coupled plasma mass spectrometry was used to determine the concentration of Cu, Fe, Mn, Mo, and Zn in the myocardium of control subjects and patients undergoing heart transplantation or left-ventricular assist device (LVAD) implantation. We used ELISA to quantify the expression of UPRmt proteins and 4-Hydroxynonenal (4-HNE), which served as a marker of oxidative-stress-induced lipid peroxidation. Concentrations of Cu, Mn, Mo, and Zn were similar in the control and heart failure (HF) myocardium, while Fe showed a significant decrease in the HF group compared to the control. A higher cumulative concentration of Fe and Zn in the myocardium was associated with reduced age_HTx/LVAD_, which was not observed for other combinations of trace elements or their individual effects. The trace elements Cu, Mn, and Zn showed positive correlations with several UPRmt proteins, while Fe had a negative correlation with UPRmt effector protease YME1L. None of the trace elements correlated with 4-HNE in the myocardium. The concentrations of the trace elements Mn and Zn were significantly higher in the myocardium of patients with dilated cardiomyopathy than in patients with ischemic cardiomyopathy. A higher cumulative concentration of Fe and Zn in the myocardium was associated with a younger age at which patients received heart transplantation or LVAD, potentially suggesting an acceleration of HF. A positive correlation between myocardial Cu, Mn, and Zn and the expression of UPRmt proteins and a negative correlation between myocardial Fe and YME1L expression suggest that these trace elements exerted their actions on the human heart by interacting with the UPRmt. An altered generation of oxidative stress was not an underlying mechanism of the observed changes.

## 1. Introduction

Elevated concentrations of trace elements, such as Cu, Fe, Mn, and Mo can be toxic to the heart [1,2,3]. However, the mechanism(s) of their toxicity are not fully understood. A recent study showed that patients with cardiomyopathy had significantly higher concentrations of Mn and Cu in their heart tissue than individuals without cardiomyopathy, suggesting a cardiotoxicity of these trace elements [3]. An accumulation of Cu in mitochondria disrupts mitochondrial function, resulting in heart damage [4]. Trace elements are vital for numerous physiological processes in the heart. For example, Fe functions as a cofactor for various enzymes, including the antioxidant enzymes catalase and peroxidase, and is found in the mitochondrial electron transport chain [5,6,7]. The trace elements Cu, Mn, and Zn are important for antioxidant defense, acting as cofactors for different isoforms of superoxide dismutase [8,9], while Cu is essential for cytochrome c oxidase activity [10].

Mitochondrial dysfunction plays a central role in the pathogenesis of different heart diseases [11,12,13]. Dysfunctional mitochondria overproduce reactive oxygen species (ROS) that cause oxidative stress, which initiates cell death pathways including the opening of mitochondrial permeability transition pores [14,15]. The homeostasis of mitochondria is maintained by several mitochondrial quality control mechanisms, including mitochondrial unfolded protein response (UPRmt), whose effector element chaperones act by repairing misfolded proteins, such as 10 kDa heat shock protein, mitochondrial (HSP10), 60 kDa heat shock protein, mitochondrial (HSP60), stress-70 protein, and mitochondrial (mtHSP70/HSPA9), while proteases act by removing proteins beyond repair like ATP-dependent Clp protease proteolytic subunit, mitochondrial (CLPP), serine protease HTRA2, mitochondrial (HTRA2), Lon protease homolog, mitochondrial (LONP1), metalloendopeptidase OMA1, mitochondrial (OMA1), mitochondrial inner membrane m-AAA protease component paraplegin (SPG7), and ATP-dependent zinc metalloprotease YME1L (YME1L) [16]. Impaired mitochondrial quality control leads to the accumulation of dysfunctional mitochondria, increased oxidative stress, and reduced ATP production, exacerbating cardiac dysfunction [17]. We recently found that a large number of genes related to the UPRmt, including regulatory elements and effector proteases and chaperones, are downregulated in patients with heart failure (HF) [18].

Here, we investigated whether concentrations of the trace elements Cu, Fe, Mn, Mo, and Zn in failing human hearts are associated with heart transplantation or the LVAD implantation recipient’s age (age_HTx/LVAD_), the expression of several effector UPRmt elements, and myocardial oxidative stress.

## 2. Results

### 2.1. Concentrations of Essential Trace Elements in Healthy and Heart-Failure Myocardium

**Figure 1:**
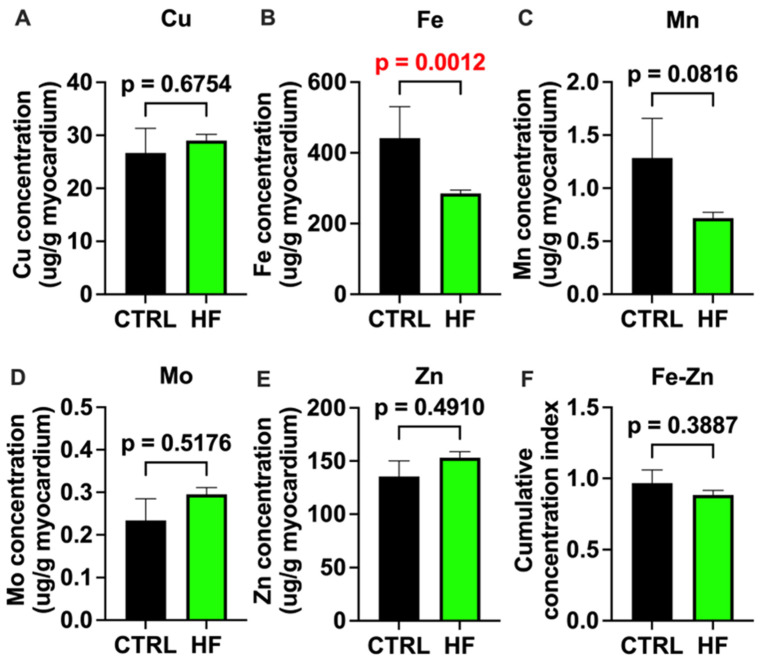
Trace element concentrations in human myocardium. (**A**–**E**) Concentrations of trace elements Cu, Fe, Mn, Mo, and Zn in the myocardium of healthy human hearts (CTRL; N = 6) and in hearts of patients with heart failure (HF; N = 126–128) are presented as summary data. (**F**) Shown are summary data of cumulative Fe and Zn concentration indexes for CTRL and HF groups. Cumulative Fe and Zn concentration index represents combined concentration of Fe and Zn in each sample. Please see Section 4 for detailed description of the calculation of the cumulative concentration index. Data are means ± SEM. *p* < 0.05 was considered significant and is shown in red font.

There were no statistically significant differences in the myocardial concentrations of Cu, Mn, Mo, and Zn between the controls and HF patients, although we observed a trend (*p* = 0.08) for a decreased Mn concentration in the HF group (Figure 1). On the other hand, the myocardial concentration of Fe was significantly lower in the HF than in the control hearts.

### 2.2. Cumulative Concentration Index of Iron and Zinc and the Patient’s Age of Receiving Heart Transplantation or LVAD

**Figure 2:**
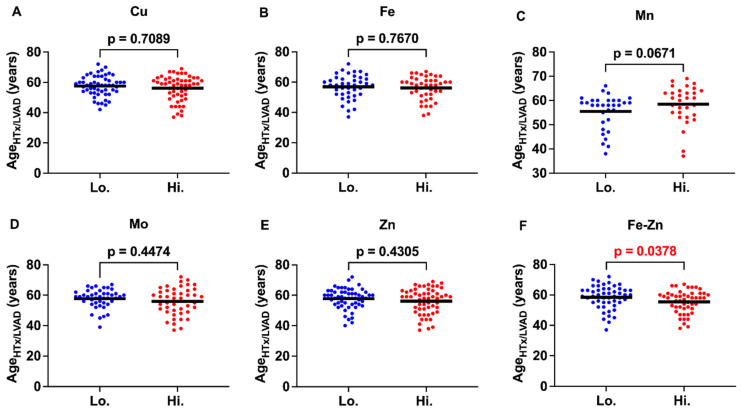
Myocardial concentrations of trace elements and the age at which patients received heart transplantation or LVAD. (**A**–**E**) Mean values (indicated by a horizontal line) and individual data points of the age when patients received heart transplantation or LVAD (age_HTx/LVAD_) are presented. Based on the myocardial concentration of each trace element, subjects were divided into groups with higher (Hi.) and lower (Lo.) concentrations. Groups sizes (N) were as follows: Cu (Lo. = 56, Hi. = 56), Fe (Lo. = 42, Hi. = 42), Mn (Lo. = 32, Hi. = 31), Mo (Lo. = 43, Hi. = 44), Zn (Lo. = 56, Hi. = 56), and Fe-Zn (Lo. = 50, Hi. = 49) (**F**) Mean values (indicated by a horizontal line) and individual data points of the age_HTx/LVAD_ in subjects with higher and lower cumulative Fe and Zn concentration indexes. This index represents the combined concentration of Fe and Zn in each sample. Please see Section 4 for a detailed description of its calculation. *p* < 0.05 was considered significant and is shown in red font.

In this analysis, the HF samples were divided into two groups; the first contained half of the samples with the highest concentrations of the individual trace elements or their cumulative concentrations, and the second contained half of the samples with the lowest concentrations of the individual trace elements or their cumulative concentrations. Since this study aimed to analyze potential toxic effects of elevated concentrations of trace elements, all samples with trace element concentrations below the control were excluded, because a deficiency in trace elements may elicit different deleterious actions. We analyzed the age of the patients when they received heart transplantation or LVAD (age_HTx/LVAD_) as an indication of HF progression. Compared to the samples with lower concentrations of the respective trace elements, the samples with higher concentrations of Cu, Fe, Mo, and Zn had an age_HTx/LVAD_ reduced by 1.4, 0.74, 1.72, and 1.62 years, respectively, but these differences were not statistically significant (Figure 2). The trace element Mn showed an opposite trend (not significant, *p* = 0.067), and the group with a higher concentration had a greater age_HTx/LVAD_ than the group with a lower concentration by almost 3 years. The group with a higher cumulative concentration index of Fe and Zn (a value representing the “combined” concentration of Fe and Zn) had a significantly lower age_HTx/LVAD_ by more than 3 years than the group with a lower cumulative concentration index of Fe and Zn. This suggests that an increased concentration of Fe and Zn together may exert cardiotoxic actions. No other combination of trace elements showed a significant difference in the age_HTx/LVAD_ between the groups with higher and lower cumulative concentrations. 

### 2.3. Correlations between Myocardial Trace Elements and the Expression of UPRmt Proteins

**Figure 3:**
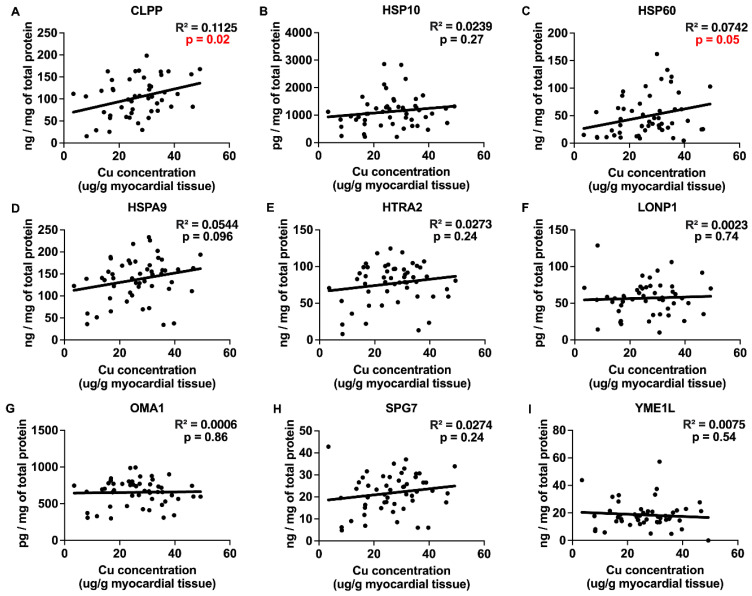
Correlations between Cu concentration in human HF myocardium and UPRmt protein expression. (**A**–**I**) Correlations between Cu concentration and mitochondrial unfolded protein response (UPRmt) protein expression in the myocardium of patients with heart failure. Included UPRmt proteins are as follows: CLPP (N = 49), HSP10 (N = 53), HSP60 (N = 52), HSPA9 (N = 53), HTRA2 (N = 53), LONP1 (N = 50), OMA1 (N = 52), SPG7 (N = 53), and YME1L (N = 53). Results of the simple linear regression are shown. *p* < 0.05 indicates significant correlations that are marked by red font. Abbreviations: CLPP = ATP-dependent Clp protease proteolytic subunit, mitochondrial; HSPA9 (mtHSP70) = stress-70 protein, mitochondrial; HSP10 = 10 kDa heat shock protein, mitochondrial; HSP60 = 60 kDa heat shock protein, mitochondrial; HTRA2 = serine protease HTRA2, mitochondrial; LONP1 = Lon protease homolog, mitochondrial; OMA1 = metalloendopeptidase OMA1, mitochondrial; SPG7 = mitochondrial inner membrane m-AAA protease component paraplegin; YME1L = ATP-dependent zinc metalloprotease YME1L.

**Figure 4:**
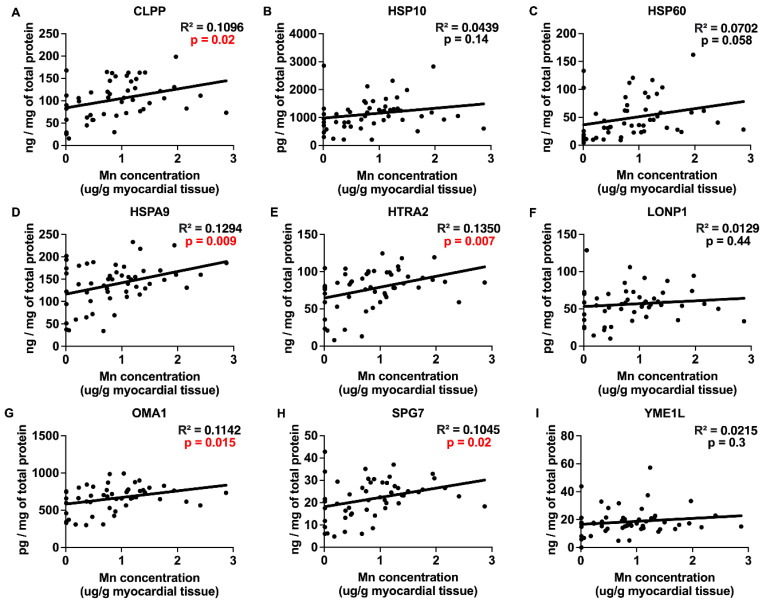
Myocardial Mn concentration and UPRmt protein expression correlations. (**A**–**I**) Concentration of Mn in individual samples of myocardium of patients with heart failure plotted against the expression of effector proteins of mitochondrial unfolded protein response (UPRmt). Proteins of UPRmt are as follows: CLPP (N = 49), HSP10 (N = 53), HSP60 (N = 52), HSPA9 (N = 53), HTRA2 (N = 53), LONP1 (N = 50), OMA1 (N = 52), SPG7 (N = 53), and YME1L (N = 53). Results of the simple linear regression are shown. *p* < 0.05 indicates significant correlations, which are marked by the red font. Abbreviations: CLPP = ATP-dependent Clp protease proteolytic subunit, mitochondrial; HSPA9 (mtHSP70) = stress-70 protein, mitochondrial; HSP10 = 10 kDa heat shock protein, mitochondrial; HSP60 = 60 kDa heat shock protein, mitochondrial; HTRA2 = serine protease HTRA2, mitochondrial; LONP1 = Lon protease homolog, mitochondrial; OMA1 = metalloendopeptidase OMA1, mitochondrial; SPG7 = mitochondrial inner membrane m-AAA protease component paraplegin; YME1L = ATP-dependent zinc metalloprotease YME1L.

**Figure 5:**
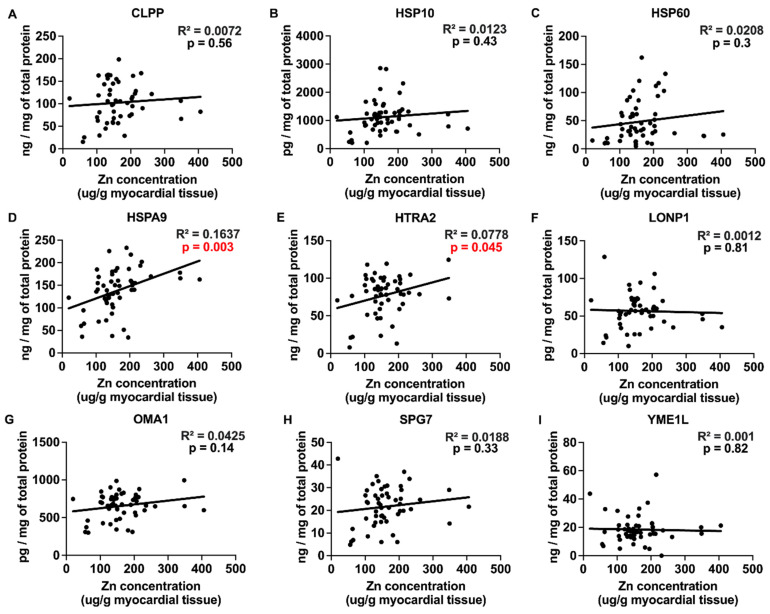
Correlations between myocardial Zn and UPRmt protein expression. (**A**–**I**) Individual data points and correlations between myocardial Zn concentration and the expression of mitochondrial unfolded protein response (UPRmt) proteins in myocardium of patients with heart failure. Proteins of UPRmt include the following: CLPP (N = 49), HSP10 (N = 53), HSP60 (N = 52), HSPA9 (N = 53), HTRA2 (N = 53), LONP1 (N = 50), OMA1 (N = 52), SPG7 (N = 53), and YME1L (N = 53). Results of the simple linear regression are shown. *p* < 0.05 indicates significant correlations, which are marked by the red font. Abbreviations: CLPP = ATP-dependent Clp protease proteolytic subunit, mitochondrial; HSPA9 (mtHSP70) = stress-70 protein, mitochondrial; HSP10 = 10 kDa heat shock protein, mitochondrial; HSP60 = 60 kDa heat shock protein, mitochondrial; HTRA2 = serine protease HTRA2, mitochondrial; LONP1 = Lon protease homolog, mitochondrial; OMA1 = metalloendopeptidase OMA1, mitochondrial; SPG7 = mitochondrial inner membrane m-AAA protease component paraplegin; YME1L = ATP-dependent zinc metalloprotease YME1L.

**Figure 6:**
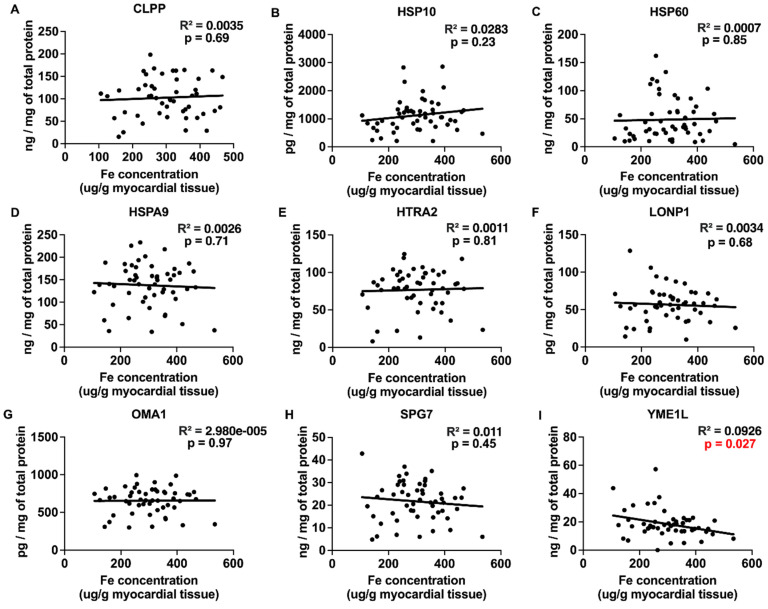
Myocardial Fe and UPRmt protein expression correlations. (**A**–**I**) The concentration of Fe in individual samples of myocardium of patients with heart failure plotted against the expression of effector proteins of mitochondrial unfolded protein response (UPRmt). Included UPRmt proteins are the following: CLPP (N = 49), HSP10 (N = 53), HSP60 (N = 52), HSPA9 (N = 53), HTRA2 (N = 53), LONP1 (N = 50), OMA1 (N = 52), SPG7 (N = 53), and YME1L (N = 53). Results of the simple linear regression are shown. *p* < 0.05 indicates significant correlations, which are marked by the red font. Abbreviations: CLPP = ATP-dependent Clp protease proteolytic subunit, mitochondrial; HSPA9 (mtHSP70) = stress-70 protein, mitochondrial; HSP10 = 10 kDa heat shock protein, mitochondrial; HSP60 = 60 kDa heat shock protein, mitochondrial; HTRA2 = serine protease HTRA2, mitochondrial; LONP1 = Lon protease homolog, mitochondrial; OMA1 = metalloendopeptidase OMA1, mitochondrial; SPG7 = mitochondrial inner membrane m-AAA protease component paraplegin; YME1L = ATP-dependent zinc metalloprotease YME1L.

**Figure 7:**
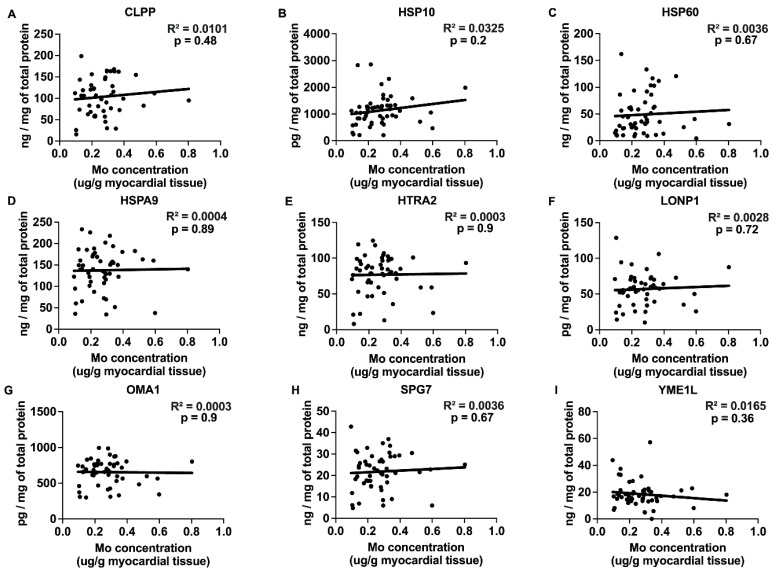
Correlations between Mo concentration and UPRmt protein expression. (**A**–**I**) The myocardial concentration of Mo plotted against the expression of mitochondrial unfolded protein response (UPRmt) effector proteins in myocardium of patients with heart failure. Analyses include following UPRmt proteins: CLPP (N = 49), HSP10 (N = 53), HSP60 (N = 52), HSPA9 (N = 53), HTRA2 (N = 53), LONP1 (N = 50), OMA1 (N = 52), SPG7 (N = 53), and YME1L (N = 53). Results of the simple linear regression are shown. Abbreviations: CLPP = ATP-dependent Clp protease proteolytic subunit, mitochondrial; HSPA9 (mtHSP70) = stress-70 protein, mitochondrial; HSP10 = 10 kDa heat shock protein, mitochondrial; HSP60 = 60 kDa heat shock protein, mitochondrial; HTRA2 = serine protease HTRA2, mitochondrial; LONP1 = Lon protease homolog, mitochondrial; OMA1 = metalloendopeptidase OMA1, mitochondrial; SPG7 = mitochondrial inner membrane m-AAA protease component paraplegin; YME1L = ATP-dependent zinc metalloprotease YME1L.

The trace elements Cu, Mn, and Zn exhibited significant positive correlations with the expression of UPRmt effector chaperones and proteases (Figure 3, Figure 4 and Figure 5 and Table 1), Fe showed a negative correlation (Figure 6), and Mo displayed no significant correlations (Figure 7). Specifically, we observed significant positive correlations for Cu as follows: Cu-CLPP and Cu-HSP60 (Figure 3); for Mn as follows: Mn-CLPP, Mn-HSPA9, Mn-HTRA2, Mn-OMA1, and Mn-SPG7 (Figure 4); and for Zn as follows: Zn-HSPA9 and Zn-HTRA2 (Figure 5). A significant negative correlation for Fe was observed for Fe-YME1L (Figure 6). We also observed trends for a positive correlation for Cu as follows: Cu-HSPA9 (Figure 3); and for Mn as follows: Mn-HSP60 (Figure 4). Interestingly, HSPA9 appeared to be positively correlated (either significantly or as a trend) with all three of the trace elements that displayed positive trends, while HSP60 and HTRA2 were correlated with two trace elements, suggesting that these three UPRmt elements could be a common mechanism of interaction between Cu, Mn, and Zn and UPRmt. We also performed a multivariable regression analysis, where the independent variables were the trace elements, age_HTx/LVAD_, and gender, and the dependent variables were the nine tested UPRmt proteins (Table 2). Similar to the simple linear regression above, the following two variables displayed significant positive correlations: Mn-HTRA2 and Zn-HSPA9, while Fe-YME1L showed a significant negative correlation. Positive correlation trends were observed between Mn-HSP60 and Mn-HSPA9, which were significant in the simple linear regression analysis. A negative correlation trend was observed for Fe-HSPA9. Mn-HSP10 and Zn-OMA1 displayed significant positive correlations that were not identified by the simple linear regression analysis. Overall Cu, Mn, and Zn showed positive correlations and Fe showed negative correlations with the UPRmt proteins, similar to the linear regression analysis. The age_HTx/LVAD_ variable showed statistically significant associations with CLPP and SPG7. Patients’ sex was also found to have significant associations with CLPP, HSP60, and LONP1. Protein HSPA9 showed both significant correlations with Zn and age_HTx/LVAD_, but the standardized coefficient for Zn (0.4323) was greater than that for age_HTx/LVAD_ (0.3264), indicating that the Zn levels had a stronger relative influence on the HSPA9 expression compared to age_HTx/LVAD_.

### 2.4. Trace Elements and Oxidative Stress by 4-HNE Detection in the Myocardium of Failing Hearts

**Figure 8:**
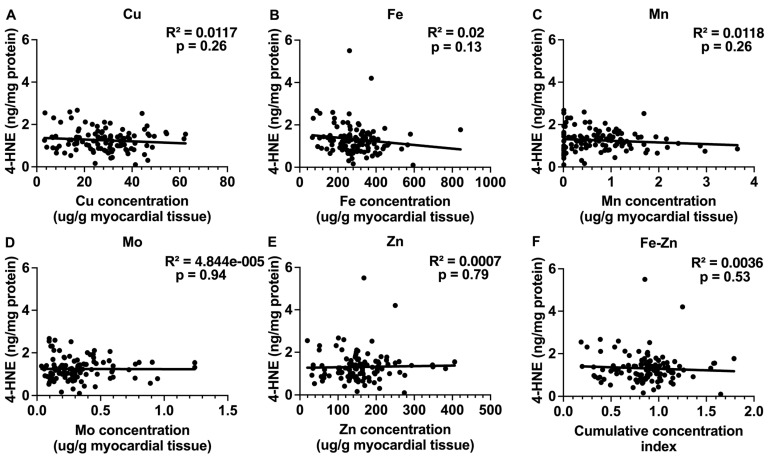
Correlations between oxidative stress assessed by 4-HNE detection and concentrations of trace elements. (**A**–**E**) Correlations between myocardial Cu, Fe, Mn, Mo, and Zn concentrations and oxidative-stress-induced lipid peroxidation in myocardium of patients with heart failure (N = 110–113). Oxidative stress was assessed by quantifying 4-Hydroxynonenal (4-HNE) with ELISA. (**F**) Correlation between cumulative Fe-Zn concentration index in the myocardium and 4-HNE levels. This index represents the combined concentration of Fe and Zn in each sample. Please see Section 4 for detailed description of its calculation. Results of the simple linear regression are shown.

Myocardial oxidative stress was investigated indirectly by quantifying the lipid peroxidation byproduct 4-HNE in the heart tissue. None of the tested trace elements nor the Fe-Zn combination showed a significant correlation with the 4-HNE levels in the myocardium of failing human hearts (Figure 8). This suggests that oxidative stress was not an underlying factor in the observed changes in the age that the patients received heart transplantation or LVAD or the expression of UPRmt effector proteins in the patients with failing hearts.

### 2.5. Trace Element Concentrations in ICM and DCM

**Figure 9:**
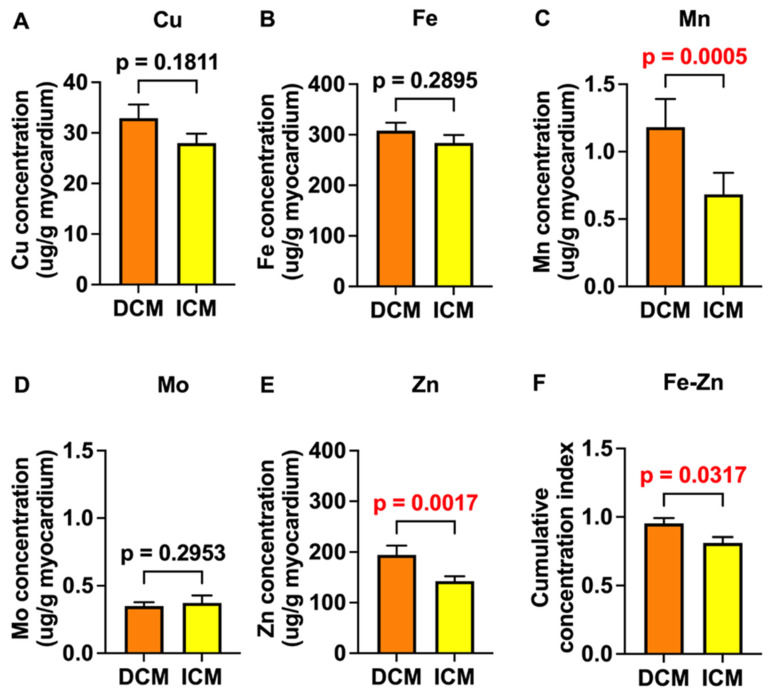
Concentrations of individual tested trace elements and Fe-Zn combination in the myocardium of patients with ICM and DCM. (**A**–**E**) Trace element concentrations in the myocardium of patients with dilated (DCM; N = 55) and ischemic (ICM; N = 59–61) cardiomyopathy. (**F**) Myocardial cumulative concentration index of Fe and Zn in myocardium of ICM and DCM groups. This index represents the combined concentration of Fe and Zn in each sample. Please see Section 4 for detailed description of its calculation. Data are means ± SEM. *p* < 0.05 was considered significant and is presented in red font.

The HF samples were divided into ICM and DCM groups for the analysis of trace element concentrations (Figure 9). When the individual trace elements were analyzed, both Zn and Mn showed a statistically significant difference and displayed higher myocardial concentrations in the DCM group than in the ICM group. There was no statistically significant difference between the ICM and DCM groups in terms of the Cu, Fe, and Mo concentrations. The cumulative concentration of Fe and Zn was also significantly higher in the DCM group than in the ICM group.

## 3. Discussion

The analyses of the myocardial concentrations of Cu, Mn, Mo, and Zn indicated that none of them had significantly different concentrations between the HF patients and the control subjects. Only the concentration of Fe in the myocardium showed a statistically significant decrease in the HF patients. There were no significant differences in age_HTx/LVAD_ when higher and lower trace element concentrations in the heart were compared. However, an elevated cumulative concentration of Fe and Zn was associated with a significantly lower age_HTx/LVAD_ by more than 3 years compared to their lower cumulative concentration. Positive significant correlations were observed between the individual myocardial Cu, Mn, and Zn concentrations and the expression of several UPRmt proteins, while Fe displayed a negative correlation with YME1L expression. Alone or in combination, the trace elements did not display a statistically significant correlation with myocardial oxidative stress, as assessed by 4-HNE staining. Zn and Mn were the only trace elements that were significantly higher in the DCM than in the ICM group.

We did not observe any differences in the myocardial concentrations of Cu, Mn, Mo, and Zn between the HF group and the control group. Only Fe showed a significant decrease in the HF group, which is in agreement with previous observations that up to 50% of patients with HF have Fe deficiency [19]. This can be explained by different mechanisms, including reports showing that mucosal edema and reduced blood flow hinder iron absorption in patients with heart insufficiency [20].

We observed that the patients with higher cumulative myocardial concentrations of Fe and Zn reached the terminal stage of HF earlier (indicated by a 3-year-lower age_HTx/LVAD_) than the patients with lower cumulative Fe-Zn concentrations. We did not observe that either Fe or Zn alone were significantly associated with the altered age_HTx/LVAD_. The association between the myocardial cumulative Fe-Zn concentration and the reduced age_HTx/LVAD_ suggests the possibility that Fe and Zn may produce an additive toxic effect on the human heart. An elevated deposition of Fe, as in hemochromatosis, is known to be cardiotoxic by inducing ROS generation, which leads to cardiomyopathy [2]. However, our study suggests that Fe could be cardiotoxic (in combination with Zn) in patients with HF even when its concentrations are not elevated. Thus, our observation that the Fe concentration was lower in the HF samples than in the control heart, could be beneficial for patients, as it would limit potential Fe-Zn cardiotoxicity. This result should be interpreted with caution, because it is known that sideropenic anemia may increase mortality risk in HF patients. The inclusion of Cu, Mn, and Mo in the cumulative concentration analysis did not result in a significant association with age_HTx/LVAD_. This implies that a moderate increase in myocardial concentrations of Cu, Mn, and Mo, as observed in our study, may not result in additional toxicity.

Here, we investigated the association between the myocardial expression of key effector proteins of UPRmt (activated by a mitochondrial damage [16]) and the myocardial concentration of trace elements, because the accumulation of several trace elements like Fe and Cu can lead to mitochondrial damage [21,22]. The analysis of the correlation between UPRmt protein expression and the myocardial concentrations of three out of the five tested trace elements (Cu, Mn, and Zn) revealed positive correlations with several UPRmt proteins. Bearing in mind that mitochondrial damage triggers UPRmt, these results suggest that Cu, Mn, and Zn may induce several UPRmt elements indirectly by causing mitochondrial damage. In contrast, Fe showed a negative correlation with the UPRmt protein YME1L. Considering that UPRmt is a stress-induced adaptive/compensatory response to impaired mitochondrial proteostasis [16], our observation suggests the possibility that increased concentrations of Cu, Mn, and Zn may disturb mitochondrial function and induce several elements of UPRmt. This is supported by evidence from the literature demonstrating the toxicity of these three trace elements [23,24]. Chaperone HSPA9 was significantly correlated with both Mn and Zn, potentially reflecting a common response element. However, the observed decrease in the expression of the UPRmt protein YME1L with elevated myocardial Fe indicates a different mechanism of action of Fe, i.e., it indicates that an increasing Fe concentration can suppress YME1L expression, a UPRmt protease known for its cardioprotective actions [25]. Thus, our study suggests that suppressed YME1L expression may be one of the deleterious effects of Fe in the heart. No correlation was observed between Mo and UPRmt protein expression. The results of the simple linear regression were mostly confirmed by the multivariable regression analysis. The age_HTx/LVAD_ and patient’s sex variables were also correlated with the expression of several UPRmt proteins. UPRmt proteases such as LONP1, YME1L, and CLPP are responsible for the removal of misfolded proteins [16]. For instance, Lonp1 protects cardiomocytes from ischemia–reperfusion injury [26], while the deletion of Yme1l causes HF [25]. The deletion of Clpp in the hearts of DARS2-deficient animals contributes to the development of cardiomyopathy [27]. Furthermore, a missense mutation causing the loss of HtrA2 protease activity in mice results in the accumulation of misfolded and damaged proteins within mitochondria, leading to mitochondrial dysfunction and HF [28]. Heat shock proteins, such as HSP60, are also cardioprotective [29], and a deficiency in HSP10 is associated with cardiac diseases [30].

We did not observe a correlation between oxidative stress, as assessed by 4-HNE detection, and the individual trace elements nor their cumulative combination. 4-HNE is one of the byproducts of the lipid peroxidation of cell membranes [31]. Since lipid peroxidation is induced by several ROS molecules, it is considered an indirect marker of oxidative stress. Although the majority of studies that have investigated trace element deficiency have found a negative correlation with ROS generation [32,33], it is reported that high trace element concentrations can increase ROS production [34,35]. Excess Zn ions have a limited ability to bind to metallothionein, thus inducing oxidative stress [35], and elevated intracellular Fe also induces ROS production, leading to a unique form of cell death called ferroptosis [36]. It seems, based on these findings and our results, that moderate changes in trace element concentrations do not play a significant role in oxidative stress and that the observed alterations in age_HTx/LVAD_ and UPRmt protein expression were independent of oxidative stress.

We observed that the concentrations of Zn and Mn and the Zn-Fe cumulative concentration were significantly higher in the DCM group compared to the ICM group. To our knowledge, this is the first study to show that these trace elements were in greater concentrations in DCM than in ICM. Thus, our study suggests the potential roles of Zn, Mn, and Zn-Fe cumulatively in the pathogenesis of DCM.

Most of the studies that have investigated the relationship between trace elements and heart diseases have focused on analyzing their concentrations in the blood or urine. A negative correlation between essential trace element concentrations in the blood and HF was reported [36]. However, several factors could interfere with this finding. A large number of HF patients use angiotensin-converting enzyme inhibitors or diuretics, such as loop or thiazide diuretics, which could lower the blood Zn concentration, potentially causing discrepancies between its concentration in the blood and tissues like the heart [37]. In patients with cardiovascular diseases, different factors could cause a redistribution of trace elements from the blood to tissues, making it even more difficult to extrapolate their blood concentrations to myocardial concentrations [33,38,39]. To our knowledge, our study is the first to demonstrate the potential cardiotoxic effect of Zn in humans, which is manifested in combination with Fe.

Trace elements may elicit different mechanisms of cardiotoxicity. The induction of cardiac fibrosis that impairs diastolic heart relaxation is reported in an overload of Fe [40] and Cu [41]. Excessive concentrations of Fe [24], Cu [24], Mn [24], and Mo [1] can also stimulate pro-inflammatory processes known to promote the development of cardiomyopathy [42]. The toxicity of trace elements, such as Mn and Fe [43], and heavy metals like Cd [44] is associated with an impaired differentiation of cardiac cells that relies on the proper induction of cardiomyogenic genes [45,46], ultimately resulting in developmental heart disorders [47]. The toxicity of Zn is related to oxidative stress, the dysregulation of Ca, the depletion of glutathione, and reduced Cu absorption [23,48].

Our study suggests that clinicians should monitor Fe and Zn concentrations in patients with heart insufficiency to eliminate their contribution to the advancement of HF, especially in those with DCM, since the Zn concentration and Fe-Zn cumulative concentration were higher in the DCM group than in the ICM group in this study. Concentrations of Fe and Zn could be assessed in the blood or, even better, by myocardial biopsy.

This study has several limitations. It is a cross-sectional study, and the observed associations cannot unequivocally indicate that trace elements cause earlier HF or induce the expression of certain UPRmt proteins. However, based on the other data in the literature showing the toxicity of these trace elements and their ability to impair mitochondrial function, such a conclusion is plausible. Additionally, the relatively limited number of myocardial samples, especially the six healthy control samples (only used in Figure 1), leads to reduced statistical power, and this may have hindered the identification of some significant associations whose effects were not large. Thus, the results from Figure 1 should be analyzed with caution. A small number of control samples was used because of the difficulty in obtaining a healthy human myocardium in a way that is appropriate for experimental use. Although our results suggest that the excessive accumulation of Zn and Fe could be considered as a significant risk factor for the acceleration of HF in patients, more studies are required to investigate the causality between the Fe-Zn cumulative concentration and the progression of HF.

## 4. Materials and Methods

### 4.1. Human Heart Samples

Myocardial samples were obtained from a transmural section of the left ventricles in control heart samples (6 samples) without known cardiac disease, provided by the National Disease Research Interchange (Philadelphia, PA, USA), and failing hearts acquired during heart transplantation or left-ventricular assist device (LVAD) implantation at the University Hospital Center Zagreb and Dubrava Clinical Hospital (128 samples). Congenital structural heart abnormalities, known genetic heart diseases, and an age below 37 years as a potential indication of a genetic heart disease were the exclusion criteria [49]. All patients included in the study gave informed consent, and we followed the principles of the Declaration of Helsinki. Prior to heart transplantation or LVAD implantation, an investigator approached the potential donors of myocardia, explained the goals of the study, potential risks, and benefits for the participants, and obtained a written consent from individuals who agreed to donate myocardia for the study. The Ethical Committees of the University of Zagreb School of Medicine (approval number: 380-59-10106-18-111/55), University Hospital Center Zagreb (approval number: 02/21 AG), and Clinical Hospital Dubrava (approval number: 2017/2310-05) approved the study. Patients with HF, defined by a left-ventricular ejection fraction below 35%, were subdivided into the ischemic cardiomyopathy (ICM) and the dilated cardiomyopathy (DCM) groups. A verified coronary artery disease was used to define the ICM. The absence of coronary disease, an uncontrolled arterial hypertension, and a significant valvular heart disease in patients with increased left-ventricular dimensions and a significantly reduced left-ventricular ejection fraction were the criteria for the DCM. Table 3 shows the important clinical parameters of the patients included in this study.

### 4.2. Analysis of Trace Element Concentrations in Human Myocardium

After drying heart tissue samples in a thermostat at 40 °C, they were weighed and digested with suprapur nitric acid (7.5 mL), puriss hydrochloric acid (2.5 mL), and puriss perchloric acid (1 mL) in a Berghof speedwave XPERT Microwave Digestion System DAK-100X (Berghof Products+ Instruments GmbH, Eningen unter Achalm, Germany). The digested samples were diluted to 50 mL with deionized water. After submerging all laboratory glassware in a HNO_3_ solution (1%) for 24 h, the samples were rinsed with deionized water. Inductively coupled plasma mass spectrometry (ICP-MS) with an Agilent 8900 ICP-MS Triple Quadrupole (Agilent Technologies, Santa Clara, CA, USA) device was used to detect trace elements in the samples. A solution containing 30 µg/L of Y, Ge, In, and Tb was used as the internal standard.

### 4.3. Calculation of the Cumulative Concentration Index of Trace Elements

To evaluate the cumulative effects of trace elements, their concentrations were standardized by calculating the “cumulative concentration index”. This represents the “combined” concentration of Fe and Zn. This factor was determined for each trace element in each sample as the ratio between its concentration in the sample and the average concentration in the control samples. The cumulative concentration index of the trace elements was then determined using the geometric mean of the sum of these concentration factors. A previously published formula was modified for this calculation [50].

### 4.4. Quantification of Myocardial UPRmt Protein Expression and 4-HNE by ELISA

Samples of approximately 500 mg of the left-ventricular myocardium that were frozen in liquid nitrogen were pulverized using a pestle and mortar. The pulverized tissue was mixed with a tissue protein extraction reagent (T-PER, Thermo Scientific, Waltham, MA, USA) containing a protease inhibitor (Halt Protease Inhibitor Cocktail, Thermo Scientific, USA) at a 1:5 weight-to-volume ratio. The homogenate was vortexed, sonicated in an ultrasonic bath, and centrifugated at 4000× *g* for 15 min at 4 °C. The supernatants were aliquoted and stored at −80 °C until further analysis. Total protein levels were quantified using the Bradford method. Briefly, the samples were mixed with Bradford reagent in a tube. The mixture was incubated for 20 min at room temperature, and the absorbance was measured in cuvette at 595 nm. A standard curve was generated using bovine serum albumin (BSA, Roche, Basel, Switzerland). Commercially available enzyme-linked immunosorbent assays (ELISAs) were used to measure the concentrations of following proteins according to the manufacturer’s instructions: HTRA2 (EHHTRA2), HSP60 (EH244RB), HSP10 (abx573810), LONP1 (abx388296), OMA1 (abx381971), SPG7 (abx546984), HSPA9 (abx350060), CLPP (abx251576), and YME1L1 (abx384347) for the analysis of UPRmt element expression and the analysis of the concentration of the lipid peroxidation byproduct, 4-Hydroxynonenal (4-HNE), for the indirect assessment of oxidative stress. All ELISA kits were obtained from Abbexa (Cambridge, UK). Titration experiments with different amounts of total proteins were previously conducted for each kit to ensure that the loaded total protein level for the samples fell within the standard curve. In short, standards, diluted samples, and biotin-conjugated reagent were loaded on plate and then incubated. After that, the HRP-conjugated reagent was added, and the plate was incubated again. Unbound conjugates were removed using a wash buffer. TMB substrate was added to quantify the HRP enzymatic reaction. Absorbance was read at 450 nm.

### 4.5. Statistical Analyses

The non-parametric Mann–Whitney test was used to test significance in comparisons between the trace element concentrations in the healthy and HF hearts and in comparisons of DCM vs. ICM due to an abnormal data distribution. Student’s *t*-test was used for comparisons of age_HTx/LVAD_ and for comparisons of protein levels in the control group versus the heart failure group. Correlations between trace elements and UPRmt protein expression and between trace elements and 4-HNE were tested by simple linear regression analysis and the Pearson correlation coefficient. Multivariable regression analysis was also used, where trace elements were the independent variables and UPRmt proteins were the dependent variables. Age_HTx/LVAD_ and gender were also analyzed as independent variables. Standardized coefficients were used to compare the relative strength of the impact of different variables on the dependent variable. *p*-values below 0.05 were considered significant. All statistical analyses were conducted using the MedCalc statistical software (Version 16.4.3).

## 5. Conclusions

In conclusion, our study demonstrated a significant association between the myocardial cumulative concentration of Fe and Zn and a lower age when patients receive heart transplantation or LVAD. This may indicate accelerated HF in individuals who have elevated cumulative myocardial concentrations of Fe and Zn. The trace elements Cu, Mn, and Zn were associated with an elevated expression of UPRmt proteins, which suggests that they may impair mitochondrial proteostasis and induce several UPRmt elements. Conversely, Fe was linked to reduced YME1L expression, suggesting an inhibition of this UPRmt element, while Mo displayed no effect on UPRmt proteins. None of the trace elements were associated with myocardial oxidative stress, as assessed by 4-HNE staining, indicating that the observed associations between the trace elements and age_HTx/LVAD_ or the UPRmt elements were independent of oxidative stress. The concentrations of Mn, Zn, and Fe-Zn combined were higher in the DCM group than in the ICM group, suggesting their potential role in the pathogenesis of DCM. 

## Figures and Tables

**Table 1:** Protein levels determined by ELISA. Means± SEM and *p*-values are shown for control and heart failure groups.

Protein Name	Control Group	Heart Failure Group	*p*
CLPP	174.5 ± 24.7 ng/mg	106.4 ± 6.3 ng/mg	0.0015
HSPA9	163.3 ± 17.8 ng/mg	135.4 ± 6.3 ng/mg	0.1786
SPG7	35.2 ± 7.9 ng/mg	27.6 ± 1.2 ng/mg	0.0028
OMA1	947.2 ± 61.5 pg/mg	665.7 ± 24.2 pg/mg	0.0004
LONP1	109.5 ± 21.6 pg/mg	58.0 ± 3.3 pg/mg	<0.0001
HSP10	2590 ± 312 pg/mg	1075 ± 66 pg/mg	<0.0001
HSP60	139 ± 55 ng/mg	46 ± 4 ng/mg	<0.0001
HTRA2	77.5 ± 8.7 ng/mg	74.5 ± 3.6 ng/mg	0.0891
YME1L	20.9 ± 4.3 ng/mg	17.7 ± 1.1 ng/mg	0.4171

**Table 2:** Results of the multivariable regression analysis. For each dependent variable, the table reports the *t*-value and *p*-value of each independent variable, indicating the strength and statistical significance of these relationships. Significant results (*p* < 0.05) are highlighted green, and trends are highlighted in yellow (*p* < 0.1).

**Dependent Variables**										
**Independent Variable**	**CLPP**		**HSP10**			**HSP60**			**HSPA9**	
	***t***	***p***	***t***		***p***	***t***	***p***		***t***	***p***
Cu	1.25	0.22	−0.08		0.93	0.74	0.47		0.59	0.56
Fe	0.07	0.94	0.777		0.44	0.18	0.86		−1.86	0.07
Mn	0.53	0.6	2.24		0.03	1.738	0.09		1.893	0.06
Mo	−0.44	0.65	−0.19		0.85	−1.12	0.25		−0.84	0.41
Zn	−0.11	0.91	0.62		0.54	0.88	0.38		3.11	0.003
Age of Htx/LVAD	2.81	0.008	0.92		0.36	0.4	0.69		2.27	0.03
Sex of patients	2.40	0.02	1.47		0.15	3.50	0.001		0.68	0.50
**Dependent Variables**										
**Independent Variable**	**HTRA2**		**LONP1**		**OMA1**		**SPG7**		**YME1L**	
	***t***	***p***	***t***	***p***	***t***	***p***	***t***	***p***	***t***	***p***
Cu	0.22	0.83	0.22	0.83	−0.69	0.50	0.24	0.81	0.40	0.69
Fe	−0.36	0.72	0.14	0.89	−0.39	0.70	−1.31	0.20	−2.60	0.01
Mn	2.16	0.04	0.51	0.61	0.778	0.44	0.89	0.38	0.54	0.60
Mo	−0.87	0.39	0.27	0.79	−0.49	0.63	−0.35	0.73	−0.12	0.91
Zn	1.44	0.16	0.23	0.82	1.978	0.049	1.20	0.24	0.10	0.92
Age of Htx/LVAD	1.23	0.22	1.43	0.16	1.642	0.11	2.31	0.03	0.59	0.56
Gender	1.56	0.13	2.08	0.04	0.04	0.96	1.43	0.16	−1.23	0.23

**Table 3:** Clinical and laboratory data of patients with HF.

Number of patients	128
Male sex, n (%)	104 (81%)
Age (y), means ± SEM	57 ± 1
BMI (kg/m^2^), means ± SEM	27.0 ± 0.3
Smoking, n (%)	59 (46)
AH, n (%)	81 (63)
DM, n (%)	44 (34)
Dyslipidemia, n (%)	76 (59)
AF, n (%)	47 (36)
IHD, n (%)	63 (49)
CKD, n (%)	39 (30)
TGD, n (%)	20 (15)
EF (%) means ± SEM	25 ± 1
LVIDd (cm), means ± SEM	7.1 ± 0.1
LVIDs (cm), means ± SEM	6.3 ± 0.1

BMI—body mass index, AH—arterial hypertension, AF—atrial fibrillation, DM—diabetes mellitus, IHD—ischemic heart disease, CKD—chronic kidney disease, TGD—thyroid gland disorder, EF—ejection fraction, LVIDd—left-ventricular inner diastolic diameter, LVIDs—left-ventricular inner systolic diameter.

## Data Availability

Data are contained within the article.
